# Recurrent Isolated Oculomotor Nerve Palsy after Radiation of a Mesencephalic Metastasis. Case Report and Mini Review

**DOI:** 10.3389/fneur.2014.00123

**Published:** 2014-07-24

**Authors:** Olga Grabau, Jochen Leonhardi, Carl D. Reimers

**Affiliations:** ^1^Department of Neurology, Zentralklinik Bad Berka, Bad Berka, Germany; ^2^Institute of Diagnostic Radiology, Zentralklinik Bad Berka, Bad Berka, Germany; ^3^Neurology, Neurologie Neuer Wall, Hamburg, Germany

**Keywords:** recurrent oculomotor nerve palsy, mesencephalic metastasis, radiation, breast carcinoma, meningeal carcinomatosis

## Abstract

**Introduction:** Recurrent oculomotor nerve palsies are extremely rare clinical conditions.

**Case report:** Here, we report on a unique case of a short-lasting recurrent unilateral incomplete external and complete internal oculomotor nerve palsy. The episodic palsies were probably caused by an ipsilateral mesencephalic metastasis of a breast carcinoma and occurred after successful brain radiation therapy.

**Discussion:** While the pathogenic mechanism remains unclear, the recurrent sudden onset and disappearance of the palsies and their decreasing frequency after antiepileptic treatment suggest the occurrence of epilepsy-like brainstem seizures. A review of case reports of spontaneous reversible oculomotor nerve palsies is presented.

## Introduction

A recurrent isolated oculomotor nerve palsy is an extremely rare clinical condition that to date has been described in ophthalmoplegic migraine, possible nerve compressive conditions (ethmoidal mucocele, neurofibromatosis type 2, recurrent hemorrhage in pituitary adenoma, pseudotumor cerebri, carotid basilar anastomosis, a Reye-like syndrome), essential mixed cryoglobulinemia, diabetes mellitus, and postvaccinal. The attacks last from 24 h to 6 months (Table 1). In Tolosa–Hunt syndrome, recurrent oculomotor nerve palsies also may occur; however, they are usually accompanied by other cranial nerve palsies (1).

**Table 1:** **Spontaneously recovering recurrent oculomotor nerve palsies**.

Reference	Etiology	Number and duration of the episodes	Suspected or possible mechanisms
Barrett et al. (2)	Neurofibromatosis type 2	3: 3 months – 1 week – 6 months	Acute conduction block
Bek et al. (3)	Ophthalmoplegic migraine	Several episodes: about 3 months	Inflammatory process
Doh-ura et al. (4)	Posterior ethmoidal mucocele	6: Duration not presented	Compression or inflammatory infiltration from the mucocele
Kotwica et al. (5)	Diabetes mellitus	5: 1–3 months – several days – 8 weeks – 3 months	Not discussed
Lance and Zagami (6)	Ophthalmoplegic migraine	Case # 1: several attacks 2 days – 2 months; case # 2: not presented; case # 3: 20 days; case # 4: 3 weeks	Demyelinization or inflammation of the oculomotor nerve
Madonick and Ruskin (7)	Carotid basilar anastomosis	2: 1–2 days – 6 weeks	Pressure on the oculomotor nerve
Manzotti et al. (8)	Measles mumps rubella vaccination	2: 24–48 h – 1 week	Not presented
Mattigk and Gaida (9)	Reye-like syndrome	2: 4–8 weeks	Transient increased intracranial hypertension
McCammon et al. (10)	Pseudotumor cerebri	2: Several hours – 14 days	Raised intracranial pressure
McMillan et al. (11)	Ophthalmoplegic migraine	Case # 1: 2 months – 10 weeks; case # 2: 2–3 days – 2–3 weeks (two additional episodes; duration not presented)	Inflammatory cranial neuropathy
Messier et al. (12)	Essential mixed cryoglobulinaemia	2: 24 h – not presented	Vasculitis of the vasa nervorum
Mohanty (13)	Hemorrhage in pituitary adenoma	2: 4 days – 8 months	Reversible compression of the oculomotor nerve
Mokta et al. (14)	Neurocysticercosis at the tegmentum of the left midbrain	2: 1–4 days	Not discussed
Ramelli et al. (15)	Ophthalmoplegic migraine	2: 2 weeks – not presented	Swelling of the oculomotor nerve
Schmal and Schulz (16)	Ophthalmoplegic migraine	2: a few days – 2 weeks	Not discussed
Present case	Meningeosis carcinomatosa, mesencephalic metastasis	Several: minutes	Epilepsy-like discharges

Here, we report on the case of a 57-year-old woman presenting with repeated unilateral internal and external short-lasting oculomotor nerve palsies caused by a neoplastic brain stem process that lasted only several minutes.

## Case History

**Figure 1:**
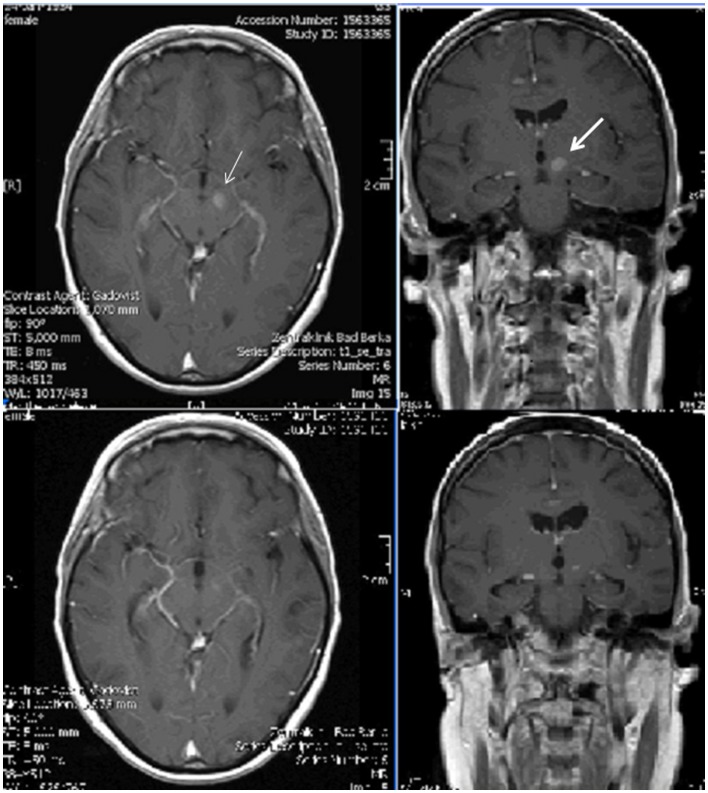
**Top: T1-weighted gadolinium-enhanced axial and coronal magnetic resonance images before brain radiation therapy: lesions on the left side of the mesencephalon (left, thin arrow) in the left thalamus (right, thick arrow); bottom: corresponding images after therapy presenting much less clear lesions**.

The female patient suffered from advanced stage breast carcinoma pT1cG3pN1, initially diagnosed 15 years ago, with metastases of the liver, soft tissues, and bone that had been treated with the administration of numerous chemotherapeutics, radiation, and chemoembolization. Finally, she developed cerebral metastases and meningeal carcinomatosis diagnosed by lumbar puncture presenting 115 leukocytes/μl (normal: ≤4/μl), increased protein content of 850 mg/l (normal: 150–450 mg/l), reduced glucose concentration of 1.8 mmol/l (normal: 2.2–4.4 mmol/l), high lactate concentration of 9.4 mmol/l (normal: 1.2–2.1 mmol/l), as well as tumor cells (Papanicolaou group V). Several supratentorial intra-cerebral contrast-enhancing lesions, which were most prominent left-sided in the mesencephalon with a diameter of 8 mm and in the left thalamus (Figure 1), were present on magnetic resonance images (MRI). Assuming the presence of cerebral metastases, a palliative radiation of the brain with 30.0 gray was performed. In addition, three cycles of 15 mg methotrexate were administered intrathecally through a Rickham reservoir.

**Figure 2:**
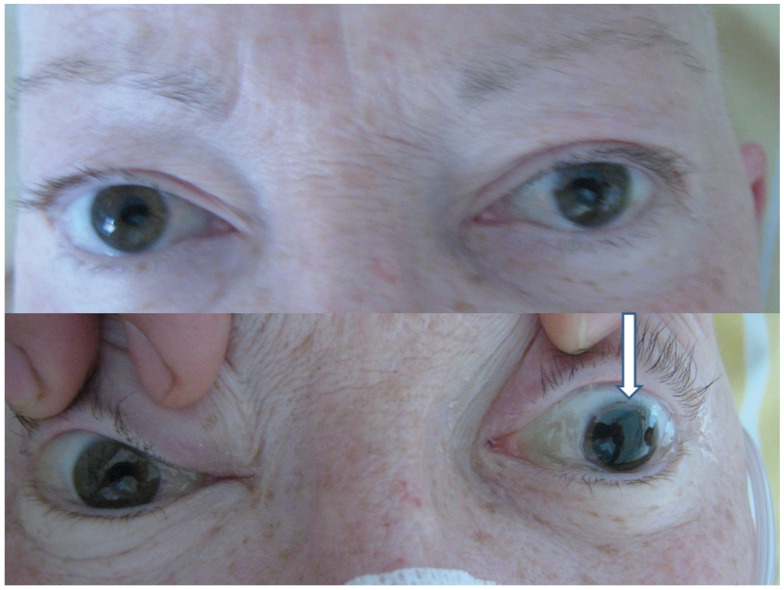
**Equal size pupils without blepharoptosis (top) in the interval between attacks of left-sided blepharoptosis, and maximally enlarged pupil during an attack (bottom, arrow) (with written permission of the patient)**.

The patient was hospitalized into the neurological department because of newly occurring headaches and temporarily reduced alertness. The initial neurological examination revealed diminished consciousness, slight neck stiffness, only small pupillary reaction of the right pupil and a large, unreactive, and maximally enlarged left pupil, a quadriparesis with only weak movements of the right arm, loss of tendon reflexes of the lower extremities, and a left-sided positive Babinski sign. During her stay, an episode of sudden complete loss of consciousness accompanied by left-sided blepharoptosis and mydriasis, and bradycardia occurred. Before and after it no weakness of the extraocular muscles, ptosis, unreactivity of the left pupil, or anisocoria were present (Figure 2). While native and gadolinium-enhanced MRI performed on the same day demonstrated an acute ischemia in the right frontal lobe, the cerebral metastases were considerably less obvious after interim brain radiation therapy (Figure 1). Assuming an atypical epilepsy-like brainstem seizure, she was treated with levetiracetam 1.5 g b.i.d. During the subsequent days, several episodes of sudden maximal mydriasis of the left pupil were observed that were occasionally accompanied by complete blepharoptosis that spontaneously disappeared after several minutes (Figure 2). During these episodes, her consciousness occasionally diminished further. Due to diminished consciousness, the oculomotor motility could not be examined properly during the attacks. In the intervals, no weakness of the extra- and intraocular muscles on either side was evident. Three electroencephalograms showed slight to pronounced general slowing down with or without few focal epileptic discharges. Lacosamide 200 mg b.i.d. was added resulting in a lower frequency of these ophthalmoplegic episodes. Furthermore, the patient became alert and orientated. However, she died 20 days after admission. An autopsy was not performed.

## Discussion

The nucleus n. oculomotorii, which innervates the lid elevators and other extraocular muscles, and the parasympathetic fibers in the Edinger–Westphal nucleus, which are responsible for the mydriasis, are located in the mesencephalon (17) where a metastasis was shown in MRI before radiation. Rarely, patients with isolated persistent oculomotor nerve palsy that was secondary to a single mesencephalic metastasis have been described, e.g., by Modi et al. (18). In another case described by Rotondo et al. (19), the oculomotor nerve palsy disappeared after microsurgical excision of the metastasis of a breast cancer. In our case, sudden onset and reversibility of the palsy due to injury of the extracerebral course of the oculomotor nerve, e.g., due to increased intracranial pressure, seems to be very unlikely. Thus, the transient malfunction of the oculomotor nerve probably must also be attributed to the mesencephalic lesion. Usually, the degree of external ophthalomoplegia is more severe than the internal weakness. In our case, obviously the Edinger–Westphal nucleus or parasympathetic fibers are more affected than the motor nucleus n. oculomotorii. To the best of our knowledge, to date, no case of recurrent oculomotor nerve palsy with sudden onset and disappearance has been published.

Neither increased intracranial pressure due to meningeal carcinomatosis, meningeal carcinomatosis itself, low intracranial pressure, which rarely induces isolated third nerve palsy (20) due to the Rickham reservoir, toxic effects of intrathecally applied methotrexate, intracranial infections such as aspergillosis (21), vasculopathy induced by brain radiation therapy or a possible aneurysma of the posterior cerebral nor a posterior communicating artery can explain the sudden occurrence and disappearance of an oculomotor nerve palsy. The unilateral affection argues against a pharmacological effect. Transient migraine-like headaches accompanied by transient focal neurological deficits (SMART syndrome) only occur several years after brain radiation therapy. Moreover, these deficits are linked to a unilateral cortical region. However, epileptic seizures, for instance, originating from the frontal lobe, may explain transient bilateral but not unilateral enlarging of the pupils. The levator palpebralis nucleus projects bilaterally and the rectus superior nucleus contralaterally. Thus, unilateral ptosis indicates that only the brainstem fascicles were affected.

The sudden onset and disappearance of the oculomotor nerve palsy as well as the observed improvement with antiepileptic drugs suggest the presence of epilepsy-like seizures that were possibly triggered by ephapses due to post-radiation scars between the mesencephalic focus and fibers of the oculomotor nerve. Possible rostral spreading of these discharges, spreading of these discharges to the formatio reticularis or additional epileptic discharges deriving from the thalamic focus may have resulted in the observed diminished consciousness.

In conclusion, the observations presented in this case report raise the question whether short-lasting cranial nerve palsies may be caused by abnormal electrophysiological discharges such as a hitherto undescribed type of brainstem seizures.

## Conflict of Interest Statement

The authors declare that the research was conducted in the absence of any commercial or financial relationships that could be construed as a potential conflict of interest.
